# Supplementary material for: FBB18 participates in preassembly of almost all axonemal dyneins independent of R2TP complex
Source: PLoS Genet. 2022 Aug 26;18(8):e1010374. doi: 10.1371/journal.pgen.1010374 (PMC9455862; doi:10.1371/journal.pgen.1010374)
Supplement: S1 Table — Immunoprecipitates with anti-GFP antibody from fbb18-1 cells expressing FBB18-YFP or wild-type cells as control were analyzed by mass spectrometry. Please note, only the subunits highlighted in red were identified both with more than 2 unique peptides and a more than two-fold increase in the spectral count (sample versus control). (DOCX) [file pgen.1010374.s001.docx]

| **Group** | **Gene/Protein** | **Gene ID** | ***Control***  Spectral count  (unique peptides) | ***FBB18:YFP-TG***  Spectral count  (unique peptides) |
| --- | --- | --- | --- | --- |
| Related to ODA | DLU1/LC1 | Cre02.g092850 | 1 (1) | 5 (3) |
|  | DLT2/LC2 | Cre12.g527750 | 1 (1) | 2 (1) |
|  | DLX1/LC3 | Cre12.g528850 | 3 (2) | 4 (3) |
|  | DLE1/LC4 | Cre01.g051250 | - | 3 (2) |
|  | DLX2/LC5 | Cre17.g714250 | - | - |
|  | DLL2/LC6 | Cre03.g187200 | - | - |
|  | DLT1/LC9 | Cre10.g428850 | 3 (1) | 5 (2) |
|  | DLL3/LC10 | Cre12.g527800 | - | 1 (1) |
|  | DOI1/Lis1 | Cre12.g552900 | 1 (1) | 2 (2) |
|  | DIC1/IC1 | Cre12.g536550 | - | 1 (1) |
|  | DIC2/IC2 | Cre12.g506000 | - | 1 (1) |
| Related to IDA | DIC3/IC140 | Cre16.g674515 | - | 8 (6) |
|  | DIC4/IC138 | Cre12.g520950 | - | 1 (1) |
|  | DII6/IC97 | Cre14.g631200 | 7 (6) | 25 (13) |
|  | DII1/p28/IDA4 | Cre12.g494800 | 2 (2) | 38 (12) |
|  | DII2/p38 | Cre03.g186300 | 6 (3) | 8 (4) |
|  | DII3/p44 | Cre08.g374650 | 19 (9) | 37 (9) |
|  | DII4/Actin | Cre13.g603700 | 84 (11) | 133 (16) |
|  | DII7/FAP120 | Cre02.g116400 | - | - |
|  | DLT3/Tctex1 | Cre01.g004250 | 1 (1) | - |
|  | DLT4/Tctex2b | Cre09.g394213 | - | - |
|  | DLE2/Centrin | Cre11.g468450 | 6 (3) | 8 (4) |
| Related to ODA/IDA | DLR1/LC7a | Cre08.g376550 | 1 (1) | 7 (2) |
|  | DLR2/LC7b | Cre12.g546400 | 1 (1) | 1 (1) |
|  | DLL1/LC8 | Cre03.g181150 | 2 (2) | 1 (1) |
