# Supplementary material for: FBB18 participates in preassembly of almost all axonemal dyneins independent of R2TP complex
Source: PLoS Genet. 2022 Aug 26;18(8):e1010374. doi: 10.1371/journal.pgen.1010374 (PMC9455862; doi:10.1371/journal.pgen.1010374)
Supplement: S2 Table — (DOCX) [file pgen.1010374.s002.docx]

| **Antibody** | **Dilution** | | **Reference or source** |
| --- | --- | --- | --- |
|  | IB | IF |  |
| Rat anti-HA, clone 3F10 | 1:1,000 | 1:50 | Roche |
| Mouse anti-α-tubulin, DM1A | 1:3,000 | 1:100 | Sigma |
| Mouse anti-IC2 | 1:20,000 | NA | Sigma |
| Mouse anti-FMG1 | 1:5,000 | NA | [1] |
| Rabbit anti-FBB18 | 1:1,000 | NA | [4] |
| Mouse anti-GST | 1:3,000 | NA | Easybio |
| Mouse anti-His | 1:3,000 | NA | Easybio |
| Mouse anti-Myc | 1:5,000 | NA | Abmart |
| Rabbit anti-DHC α | 1:1,000 | NA | [2] |
| Rabbit anti-DHC β | 1:1,000 | NA | [2] |
| Rabbit anti-Dynein b | 1:3,000 | NA | [5] |
| Rabbit anti-Dynein c | 1:5,000 | NA | [5] |
| Rabbit anti-Dynein e | 1:5,000 | NA | [3] |
| Rabbit anti-NAB1 | 1:10,000 | NA | Agrisera |

IF, immunofluorescence; NA, not applicable; IB, Immunoblot.

**References**

1. Bloodgood, R.A., and N.L. Salomonsky. 1994. The transmembrane signaling pathway involved in directed movements of Chlamydomonas flagellar membrane glycoproteins involves the dephosphorylation of a 60-kD phosphoprotein that binds to the major flagellar membrane glycoprotein. J Cell Biol. 127:803-811.

2. Fowkes, M.E., and D.R. Mitchell. 1998. The role of preassembled cytoplasmic complexes in assembly of flagellar dynein subunits. Mol Biol Cell. 9:2337-2347.

3. Kubo, T., T. Yagi, and R. Kamiya. 2012. Tubulin polyglutamylation regulates flagellar motility by controlling a specific inner-arm dynein that interacts with the dynein regulatory complex. Cytoskeleton (Hoboken). 69:1059-1068.

4. Liu, G., L. Wang, and J. Pan. 2019. Chlamydomonas WDR92 in association with R2TP-like complex and multiple DNAAFs to regulate ciliary dynein preassembly. J Mol Cell Biol. 11:770-780.

5. Yagi, T., K. Uematsu, Z. Liu, and R. Kamiya. 2009. Identification of dyneins that localize exclusively to the proximal portion of Chlamydomonas flagella. J Cell Sci. 122:1306-1314.
