# Supplementary figures and images for: FBB18 participates in preassembly of almost all axonemal dyneins independent of R2TP complex
Source: PLoS Genet. 2022 Aug 26;18(8):e1010374. doi: 10.1371/journal.pgen.1010374 (PMC9455862; doi:10.1371/journal.pgen.1010374)

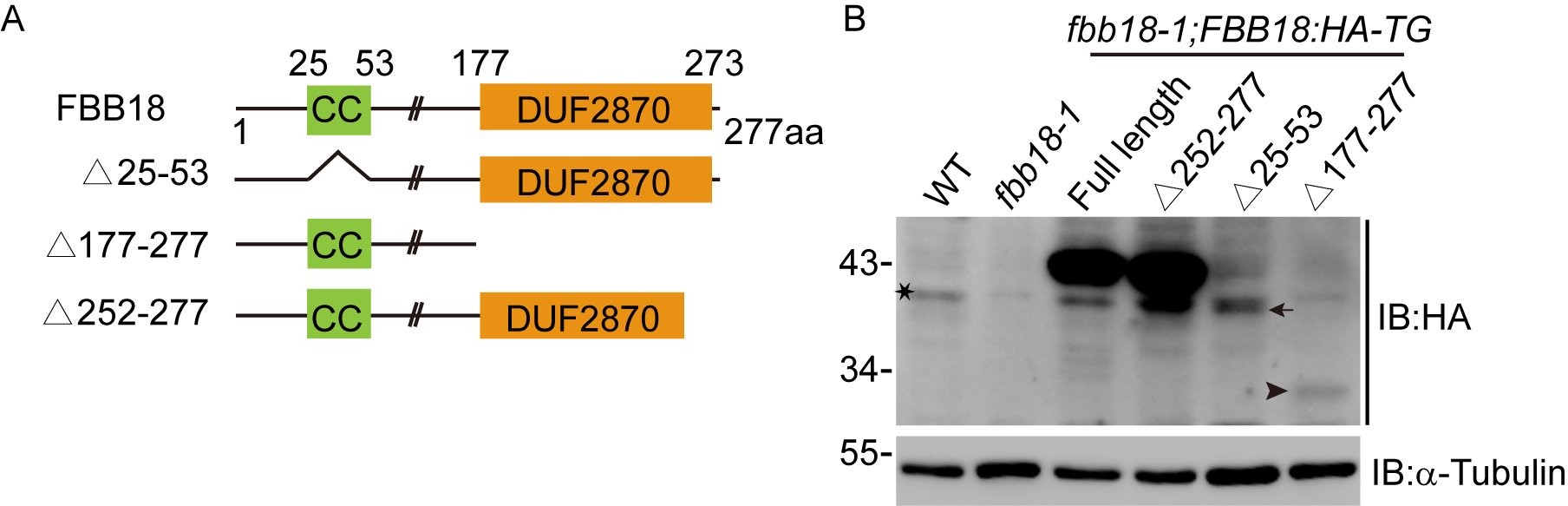

Supplement: S1 Fig — (A) Domain organization of FBB18 and its truncated proteins. The coiled-coil domain and DUF2870 are deleted in FBB18Δ25–53 and FBB18Δ177–277, respectively. FBB18Δ252–277 mimics a human mutation that results in a C-terminus truncated protein. Numbers denote the residue positions. (B) Immunoblot analysis of the expression of the deletion mutants. Deletion constructs with a wild-type construct as a control were expressed in fbb18-1 followed by immunoblotting. Wild-type (WT) and fbb18-1 cells were also used as controls. Arrow and arrowhead mark the truncated proteins FBB18Δ25–53 and FBB18Δ177–277, respectively. Band marked by asterisk indicates non-specific band. (TIF) [file pgen.1010374.s003.tif]

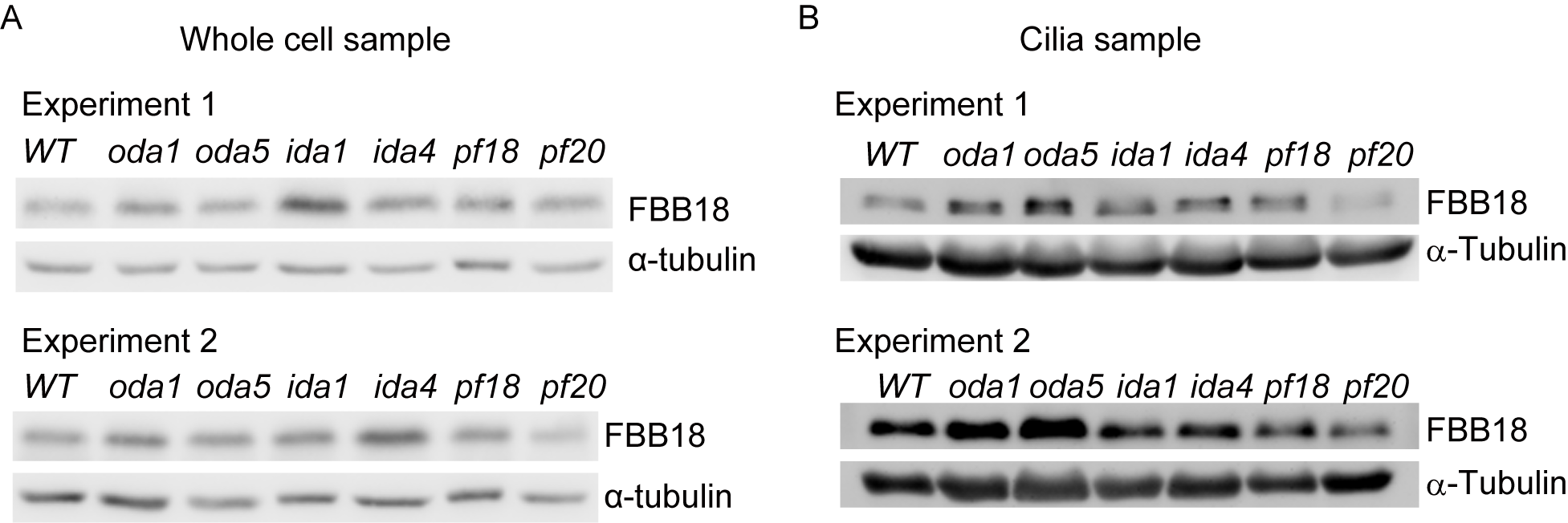

Supplement: S2 Fig — Whole cells (A) or isolated cilia (B) from cells as indicated were subjected to immunoblotting with the indicated antibodies. Please note, the ciliary abundance of FBB18 varies with the mutants. We failed to observe a correlation between the ciliary abundance of FBB18 with the phenotype of impaired cilia motility. (TIF) [file pgen.1010374.s004.tif]

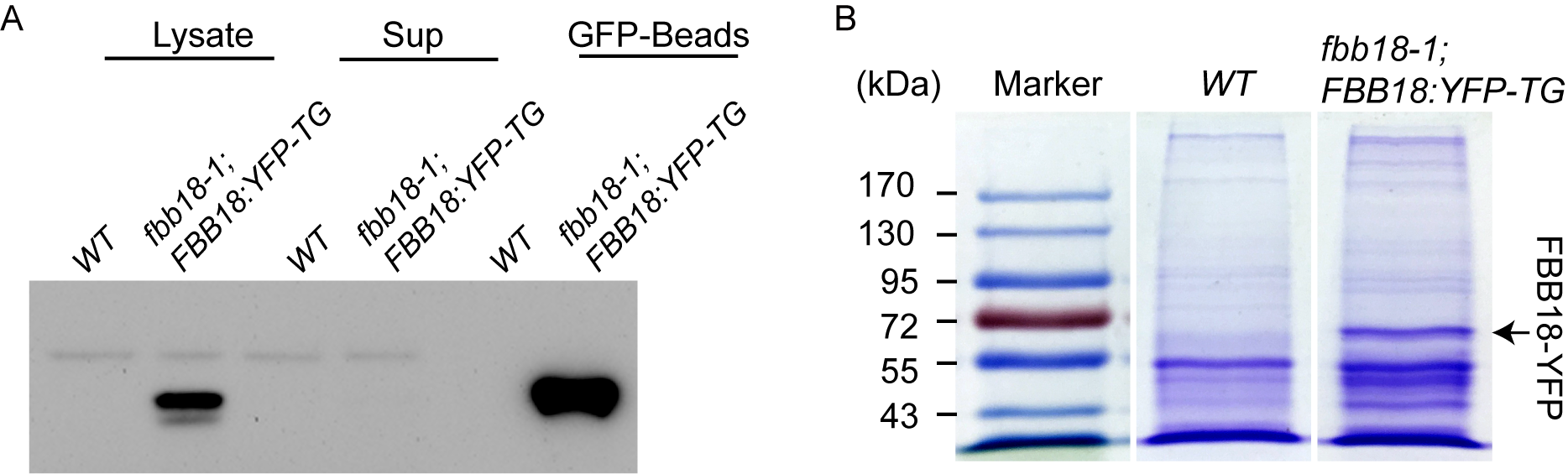

Supplement: S3 Fig — Cells expressing FBB18-YFP with wild type cells as a control were immunoprecipitated with anti-GFP antibody followed by immunoblotting with anti-GFP antibody (A) and SDS-PAGE (B). The gel slices were used for mass spectrometry analysis. (TIF) [file pgen.1010374.s005.tif]
