# Supplementary material for: FBB18 participates in preassembly of almost all axonemal dyneins independent of R2TP complex
Source: PLoS Genet. 2022 Aug 26;18(8):e1010374. doi: 10.1371/journal.pgen.1010374 (PMC9455862; doi:10.1371/journal.pgen.1010374)
Supplement: S4 Fig — Residues highlighted with red or blue are the peptides identified by mass spectrometry. (PDF) [file pgen.1010374.s006.pdf]

MSIFWEVPNAQGEAPCPRSGHSFTVLGERFVLFGGCGRKDGKAAAFNDLYEL
DTSDPDEYKWKELVVANAPPPRARHAAIALDDKRLLVFGGLNKRIRYNDVW
LFNYDDKSWTCMEVEGAAPEPRAHFTATRFGSRVFIFGGYGGSGQVYNEMW
VLHFGEDGFR**WQNITESIEGTGPAPRFDHSAFIYPVTPNSDTYDK**LLIMGGR
DLSQMYQDSHMLDLNKMAWENETQPPTLPYEICNNVCDGIESVPYHKVFSFG
GRKGMMQYLNTVEVMDCGSQMWSTPPVDHGVAPVGREDTAWVFDVKTCS
LLIFGGWANRWLGDLHKLNVSPIIGPPYACTAIQPEMGPVFGSTELVIRGLRFR
DGKVQVKFGLSEKNEVVVEGTYVDQETIR**VQTPNYEQFGALTVDVRVSING**
EGWTVNKIK**YAYFANTAAR**NCIAYGPGLLAETISGVEVPFIIQAKDTLNDKR
TSGGDVFK**VTVVSADGKNEGVSR**VRDLQNGQYEVQYAAPTAGPYLIHVAF
NELGTSDFVPIRGSPFTVKCTDSWTKHRVMGAAPAKRKGATICTMGNELVLY
GGDKSGVTVLNTEGAEWR**WSPATVSGSTPPDR**TAHSTVVLSDGELVVFGGI
NLADQNDLNDIYYLRKQGEGWVWSCPSESRPYIRHPKGAAAVSAEPSAEPAA
EPAAEPAAEPDADAPAAEPAAEGEEGAVPAEGEEGAEGASGSRPVSAKPAPA
AAAPAAEALPELPVSARNSHVAVAIDKDLYVMMGDHDGDLMTELAMVDTS
DRTCAHWLEPILKGDVPVPRKACAAAATGNTIVLFGGQTQNADGEATVTGD
LVIMEVTGPNSIQCAVNPAAPGASGSPAARYGAVMQEFSNGKLFLHGGMDA
ASKPLNDGWLFDVPSKTWQCVYVGSSDVVLPTGSLATLWRNR**IVLVSAAVG**
SPKLDSVQSLDFQELRDSVAFTPKMRASTETLLKGLEDWVDTQAHGMELA
RSPEKLSKDFENGLRKVMDALFQVKSQRSQTDLLIDQLHEAFAQLAEEKVPGI
NKMEKRLEAAAHKWDEIKKAQPQVKTDVEPIQAAKGEDIKKEIETFAAKVR
NYRADFRRRGFFKYATGFDGAYPLLDAAAHELAELKKECDRLSELASVFEFP
QAIEPVTVAIKETVEDLVMVKDVWDTAVLCELQFQDWRQTLWSDIRTDIME
EGAKQFVKEVKSLHKKVRDEDVFRGVDQVVK**NFLVSVPLVADLR**SPAMRD
RHWEQLMATTKMTFNVKDPNFKLDDLLALELHKFEEEVGEIVDRAQKEEKM
EIAIRKLNDTWTRVEFQFHRHKDYDVHTVKMAEEDFEALEDNQVQVQGMIA
NRYMATFKDEILGWQKK**LNDVADVNQIMAEIQR**TWAYLESLFIHSEEVKKE
LPQATER**FAAIDTEVKK**VLREFQQLKNCVSCCNREGLYANLETQERELEICK
KALNDYMESKRRAFPRFYFVSSADLLDILSNGNNPMRVQIHMNKCFQAIDKL
R**LDSEEVVPGR**RPKALGMESCVGIEYVPFSSPLPLENKVEQYMNDIIAKMRN
ELRMVLKASVEDYPSKPRDKWLFDWPSQIILVVNQIYWCLEVEQAFTEMARG
DKGAMSKYNEFQVKQLTKLIEVTRTDLSKPDRQKIMNMITIDAHSRDMVLAV
IEAGADQPDSFQWVSQLRSYWDRDISDCRIRICDASFPYGYEYLGNGPRLVIT
PLTDRIYITATQACWLSLGTAPAGPAGTGKTETTKDLSAQLGKSVYVFNCSPE
MDYRTMGDIFKGLAASGSWGCFDEFNRLVPEVLSVCSVQYKCVTDSQKKKT
MLPGRGLEYIKDGVKHPAVEHWSFIAADGVEMPLEEGTSAFITMNPGYIGRA
ELPESLK**ALFRPITVMVPDR**QLIMENMLMAEGFVEAKMLAKKFASLYYLLE
DLLSPQKHYDWGLRAIK**SVLVVAGSLLRAEAGQVEADVLFR**ALRDFNIPKI
LAQDMVIFMGLLNDLFPGIDPPRKR**DMEFEDVIVSTIKDLGLTPEDDFVLR**V
VQFSELLAIRHCVFLMGPTGTGRTECYRVLAKAITKGCNNPVNDYLKMTNKK
KVVIRDINPK**SISTYELYGQVNQATR**EWKDGLLSYYMRELANMPGDDPKW
LLLDGDLDANWIESMNSVMDDNR**LLTLPSNER**IRVLPHMK**LIFEIR**DLKFAT
PATATRAGILYISEGQQWHNMAMSWINRVVKPYAERAKWKDPQLPCTWLRE
MFDKYIPPTLLEMKKSYSHITPLAQMNFISTLVNIMEGVLKPENLSNKADQAM

FEMYFVFAMIWAFGGGLVEKDGIPYRRNFDKWFKQTWTTVKIPGKGTVYDY
FVNPKTQKFQPWAELVTDIDYDGSRPMSTVFVPTAETSSLRFFLDMMVDLRK
PIMFVGGAGVGKTQLVKGKLGSLNEEQISLSISFNYFTDVVSFQK**VLESPLEK**
K**AGINYGPPGTK**QLIYFVDDLNMPK**LDLYETAMPISLIR**QHLGWGHWFDR
AKLTPKNINNTQYVACMNPTAGSFIINPRLQR**LFMTLAVDFPGQDSLMKIY**
GTFLQGHLKK**FSESIQDMGTKILQAALALHDR**VSQTFRKTAINFHYEFTVR
HLANVFQGLLMSTPEAFNSPTKWGKLWLHESERVYADRLVSLYDLDAYN
KAATAIAKKYFSVADIDDYYKKKDPKPLIFCHFARGLADK**AYDEVADYTSLY**
KTLTEALNEYNETNAAMDLVLFEDAMKHVCRISR**IVSNPSGHALLVGVGGS**
GKQSLARLAAHICGYATQMIVISGSYSMNNFKEDIQKMYKRTGVKGEGVMF
LFTDSQIVDERMLVYINDLLSSGEIPDLFPQEDRDEIVNALRSETKSLGLLDTA
ENCWATFIQKVKTNLHMVFTASPVGENFRVRSQRFLATVTSTVIDWFQPWPE
SSLFSVAKRFLDEVDLGEDAVRNAVVEFMPYSFQLVNKVSIKFREQERRYNY
TTPK**TFLELIK**LYKNVLAAKRKANQDNTERLENGLHKLHKVQADVDILVEE
AKVKAVEVEHK**VASANIFAEQVGVEK**EK**VNAENAAAQVEAEK**CAVIAKEV
SEKQASCEKDLAAAEPLVAEAMAALETVTKKDLGEAKSLKKPPPGVDDITAV
VIILLENNPKDKSWQAAQKLMNNVDKFLERVKSFKSVIDAGQVARKTVDAC
RPYLALEWFNREAIGKKSAAAAGLCEWAVNIIKYYDVVQEVEPKRQELAAA
NAKLEEANVTLAAVEEKVALLNAKVQELEQQYKEANDDKEAAIRESERCQR
KLELANR**LINALASEGER**WALTVEQLRKSYEVLTGDMLLAAAFVSYAGPFT
AKFRAQLIDDWILFLRERHMPMTEGITDPLKVLVDDALVAGWIR**EGLPSDPT**
SVQNGTILTNSERWSLMMDPQLQGILWIKERESKNNLQVTRMGASNMLQV
MER**AIEAGHSVLVENMGETIDAVLNPIITR**STFKKGRSLYVKLGDKECEYN
KNFRLFLHTKLSNPHYPPEIQAETTLINFTVTEAGLEDQLLALVVNKERPDLEE
TKTQLIIQNTEFTIK**LKELEDGLLLK**LSTAEGDITEDVALIESLEDAKRVSTEIS
EKVKESRETEAAINENRNKYRTVAARGAMLFFLLNSLNKIHAFYQFSLNAFV
TVFSRGLDLAPGGRKKGKGLKKTPSLRDQPMDHQSLMEKARRSSGVGDR**RP**
SQEGLPGPEASQASLAESQGGRGSQVGDAEDEDDESFAMAPEALEQRLVN
LLETCTFTVYNYTRRGLFDRDKLIVLSLLTFTILLRSQAVDASEYEALCRGMR
NPTPPPITDDLSRWMAESQWAALDVLTTLPCFAHLAKDMEKNSDDWFNWCN
NEAAERAPMPGEWGKLTEFR**QLLIIR**ALRPDRITNALQNFCEHMMGSDYVN
QDAFSPAAMMDESSSATPIFFILFPGYSPSKEIEVYANKCGYSVANGRLCLISM
GQGQEAPAEAVLDKYTREGGWVFLDNVHLMQGWIPKLERKLEIAAESAHPD
FRCFFSAEPINGAPHANIIPESILQTCIKISNEPPSDMKSNMRRAFAAFTPEQCD
RPSTPAKRVAFRAILFGLCFYHSLLLGRKKFGVGIGTGSGSGLGFCRGYSFNIG
DLTTCGDVLYNYLEAYEQIPWRDLQYMFGEVFYGGHITDSMDRRCCTTYLE
VLIRNEILPKGNPDEVEAWEAPTLELAPGFFAPKPVDYPTLKEYIETSLPAESP
VVYGMHPNAELSLLTSLGETLFK**TVVEVAGGGGGGGGGGGGGENAVR**QA
LETFKERLPEPFNMVEVELRVKEKTPFVVVALQEATRMNALLSEMKRSMEEL
QLGLDGALNMSDNMEKLAKGIASNTVPELWMSCMSTR**VQEVYTLTAWYQ**
DVVKRHDQLSAWTAGDIITPHSVWLPGLFNPK**AFLTAVMQTFAR**ANKLPLD
VMKFMTEVTR**MTSPEQVTEAAPLGVYVHGLVLEGAR**WDREDGCLRDSKP
NELHPAMPVLQVKPVTADQFNLEGYYECPVYTNMQR**ANVYSPVVSTFTLR**T
QDMPAKWVLASVALLLQDDLAG*
