# Supplementary material for: FBB18 participates in preassembly of almost all axonemal dyneins independent of R2TP complex
Source: PLoS Genet. 2022 Aug 26;18(8):e1010374. doi: 10.1371/journal.pgen.1010374 (PMC9455862; doi:10.1371/journal.pgen.1010374)
Supplement: Attachment — Submitted filename: Response to reviewers.docx [file pgen.1010374.s010.docx]

**We are grateful to the Editor and reviewers for the constructive comments.**

**1. Response to general comments**

Nomenclature
• You need to follow the nomenclature rules at the Chlamydomonas Resource Center. For example, a transgene should be FAP298:HA-TG. You need to indicate it is a transgene and not an edited gene. <https://www.chlamycollection.org/resources/tools/gene-nomenclature/>

We have made according changes per the suggestion.

• Genes should NEVER include Cr in the name.

Changes are made.

• Do not use superscripts, but give them allele names. (see link to nomenclature)

The superscripts were used for truncated constructs, not for permanent mutants. We checked the link for nomenclature and found no rules regarding this situation. The use of superscripts is widely accepted in the field of plant and animal research. We felt that the use of superscripts will help the readers for understanding the related work.

• This gene has been named in Phytozome in 2005 and , it has been published as FBB19 (My choice for the Chlamy gene). It is also known as kurly, C21orf59 and finally as CFAP298. No matter what, it is not CFAP298.

• CFAP298 is the human protein name and not the Chlamydomonas name. You must use it, it is FBB18 or FAP298.

We think that FBB19 is a typo. Searching FBB19 in Uniprot, NCBI gene and chlamy genome revealed that it is a different gene from FBB18.

Per the suggestion, CFAP298 is revised to FBB18 when we refer to the chlamy gene while CFAP298 is used for general descriptions.

Introduction and Discussion points
• The manuscript states that DNAAF7 stabilizes IC1/IC2. The authors should look at the work from Mali (REF 47).

Indeed, we have noticed the difference between different publications. Mali et al., 2018 reported that ZMYND10 does not affect the stability of IC1/IC2. This work prevented two evidences as far as we understand: 1) co-IP, 2) input for co-IP in Figure 4. For the co-IP data, it really depends on the amounts of antibody and cell materials used, and the extent of protein degradation. If there is a 50% degradation, the amount of antibody used can only pulldown 25% of proteins, one is expected to see no difference. For the input for co-IP, there is no loading control. Thus, we think the evidences prevented there do not warrant the conclusion that the stability of IC1 and IC2 are not affected by ZMYDN10.

For the conclusion that DNAAF7 stabilizes IC1/IC2 (Cho et al., 2018), it was shown in Figure 5A that DNAI1/IC1 was reduced in the mutant with actin as a loading control. Furthermore, it showed by co-expression that ZMYDN10 can stabilize IC1/IC2.

Thus, we tend to believe that ZMYDN10 has a role in stabilizing IC1/IC2. However, in the revised text, we now acknowledge the different conclusions from different studies.

• Patients have laterality defects rather situs inversus as they are patients with heterotaxy. Laterality is a more general term.

Excellent points! Laterality is now used.

• Wild-type should have a hyphen when used as an adjective (line 125 as one example).

Excellent！ Changes are made.
• The American Society of Human Genetics recommends that changes in patients be referred as variants and not as mutations. (one example on line 171).

Excellent！ Changes are made.
• Pleases avoid flagella and deflagellation. See line 190 on page 8.

Great, deflagellation is changed to deciliation. Flagella is only used when describing sperm flagella.

Experimental Questions
• The FAP298:YFP-TG construct is never characterized but is used in several experiments. It must be characterized. The authors should at least look at cilia numbers and lengths. They should characterize at least three independent transformants.

Indeed, we have characterized. We did not show these results because we showed data on FBB18:HA. For the rescue experiments, we routinely obtained around 10% positive transformants and they showed wild-type phenotypes. We now revise the text to stress this fact (see page 11. Lines 257-258).

• The ODA and IDA protein levels should be quantitated.

The ODA and IDA protein levels in *wt* and *fbb18* mutants for Figure 2C and Figure 3E are quantitated by densitometry. The data are presented in the figure legends.

• The manuscript states that DNAAF7 stabilizes IC1/IC2. The authors should look at the work from Mali (REF 47).

Please find our explanations above in our response for first comment under “Introduction and Discussion points”

• The authors state that cilia are isolated with 0.5% Non-Idet. I would guess that they have axonemes and not cilia.

We examined our manuscript. On line 478, we say that “isolated cilia” were dissolved in 0.5% NP40 for “cilia fractionation”.

• In the previous characterization of FBB18 in the cilia, it was stated that the level is low. Please compare your results to the published data.

We compared the relative protein level in the cell body and cilia on the same immunoblot and found that FBB18 was present predominantly in the cell body (Fig. 3C). As far as we understand, in the previous publication, the protein level of FBB18 in chlamy cilia and cell body was analyzed on different blots. Thus, it would be hard to reach any conclusion about the relative distribution of FBB18 between cilia and cell body in that publication. Please notice, in the abstract of that paper, it was stated that “FBB18 is a flagellar matrx protein”. That said, we could not compare our data with the previous publication.

• The proteomics and the blots do not agree very well. Please discuss.

We did proteomic and immunoblot analysis of dynein heavy chains from whole cell extract of *wt* and *fbb18-1* cells. Because of the availability of the antibodies, we only assayed for immunoblotting three heavy chains, i.e. DHCα, DHCβ and dynein b (Figure 3E). In the mutant, DHCα and dynein b were reduced significantly while DHCβ was reduced mildly. For the proteomic assay, DHCa, dynein b and DHCb were reduced 89%, 54% and 9%, respectively. Overall the reduction is consistent for each dynein in these two datasets.

However, we agree that these two sets data do not agree well when one looks at the numbers. We think the proteomic data were more accurate since the two duplicate data are pretty close. The inconsistency between these two different assays may be affected by many factors. The immunoblotting data may be affected by factors including the sensitivity of the antibodies, cell materials loaded, and etc. The antibodies we used were gifts given to us more than five years ago. The sensitivity may be low. We have now indicated the apparent disagreement between these two types of data in the revised text.

Reviewer's Responses to Questions

**Comments to the Authors:
Please note here if the review is uploaded as an attachment.**

**2. Reviewer #1:** Defects in CFAP298 (aka C21orf59 and FBB18) have previously been shown to affect dynein assembly/ciliary motility in zebrafish, humans and planaria; human patients exhibit primary ciliary dyskinesia phenotypes. Biochemical studies using Chlamydomonas found the protein both in the cytoplasm and the ciliary matrix. In this study, the authors identify and characterize a Chlamydomonas null mutant in CFAP298. They find it is present mainly in the cytoplasm but that it is also readily detectable in cilia. The mutant exhibits no or short cilia and proteomics/pull downs identify associations with dynein heavy chains (HCs) and several other factors known to be involved in the cytoplasmic preassembly of axonemal dyneins. They find that CFAP298 associates with the RuvBL1/2 ATPases but they find no evidence for association with other components (scaffolds and PIH proteins) of R2TP complexes and its variants.

Comment to the Journal
The figures provided as part of the manuscript pdf are very low quality. I can only assume that this reflects the automated manuscript processing system that generated the pdf as the supplemental data file figures provided directly by the authors are of high quality. Providing such low quality figures to reviewers does authors a great disservice.

Comments to the Authors
1. The abstract (and many other places) should indicate that the ODA/IDA loss is partial in the cfap298 mutant and that the amounts of some major HCs are barely affected. Also, the abstract refers to “new mechanisms of axonemal dynein preassembly” but no mechanistic detail is actually provided.

Great, it is revised accordingly. Mechanisms is revised to insights.

2. The authors show that the alpha HC is missing and the amount of beta HC in cfap298 cilia is reduced somewhat. Based on this and similar data for several inner arms they state that CFAP298 is “required for the assembly of both ODA and IDA in cilia” (lines 157-158) – considering many ODA/IDAs still assemble this seems a rather strong statement as the effect is clearly not “all or none” but is rather much more nuanced.

Ok, got it. This statement is changed to “required for proper assembly…”. We hope this change would communicate our ideas more accurately.

3. The observation that assembly of the alpha HC particularly depends on the CFAP298 DUF2870 domain is interesting as the alpha HC (which is closely related to the unaffected beta HC) is unusual in having a completely different N-terminal region compared to all other Chlamydomonas HCs. Although not a requirement, it might be interesting to test whether CFAP298 interacts specifically with this alpha HC region that consists of kelch domains and several Ig folds.

Excellent points！This point is included in the revision.

4. Although CFAP298 is said to associate with the beta and gamma ODA HCs, its loss appears to have little/no effect on their cytoplasmic assembly as they are present in the mutant at ratios of 0.9 and 1 compared to wildtype (it is quite possible that the small reduction in beta HC amount might reflect the total loss of its binding partner the alpha HC rather than a direct effect of CFAP298 loss). As these (beta and gamma) are 2 of the 3 most abundant axonemal HCs in Chlamydomonas, if CFAP298 is central to HC folding/stability why are they unaffected? From my understanding, the IP (table 2) was from cell extracts and so if CFAP298 binds alpha HC (which is greatly affected by cfap298 loss), they would also IP beta and gamma which are tightly bound to alpha even though CFAP298 may not be actually binding them directly at all. Similarly, if CFAP298 associates with one HC of the I1/f dynein one might expect to pull down the second HC as well even though it may not interact directly. Thus, this needs clarifying. Care should be taken not to imply that CFAP298 is a DNAAF for all HCs, which it clearly is not. Many sentences mentioning this concept are sweepingly broad (e.g. lines 333-334 and elsewhere) and it should be made clear they do not apply to all axonemal dynein HCs equally. Perhaps it might help if the authors could identify or discuss some common feature(s) of the HCs most dramatically affected compared to those that are not. What special feature of these HCs might CFAP298 affect?

Good points! We agree that FBB18 is NOT central to folding/assembly of each HCs. It involves the preassembly of most of the HCs based on its association with the HCs and differential reduction in the abundance after its loss. In the discussion, we discussed that FBB18 may not directly interact with all of the dyneins. It may function as part of a network of DNAAFs to facilitate the folding.

Two of the dyneins only reduce around 10%, but the data from the two experiments are very close. It has been shown that loss of DHCα in oda11, there is no change in the abundance of DHCβ and DHCγ (Fowkes et al., 1998, MBC). While DHCγ has no change, thus we think the 10% reduction of DHCβ is believable.

In our IP experiments, IC1/IC2 were not enriched in the IP. Thus, we think that it is unlikely that FBB18 pulls down all of the ODA HCs because of its interaction with only one of them.

In the revision, we include the distinct feature of DHCα from DHCβ and DHCγ as suggested.
5. In the IPs (table 2), where in the HCs do the identified unique peptides derive from? Do they include C-terminal peptides in which case one might predict that CFAP298 is associating with fully formed dyneins (i.e. post chaperone requiring) – how would this affect their interpretation of CFAP298 function? Also, the authors should detail what other dynein components (if any) were found in these IPs as this again directly impacts on the state of the dyneins with which CFAP298 is associating.

Excellent points! Per the suggestion, we examined the identified peptides of DHCα because its stability is severely affected by the loss of FBB18. Several peptides are found at the C-terminus. Thus, FBB18 associates with fully formed dyneins. It will predict that FBB18 and its associated DNAAFs functions after completion of translation but we could not exclude the possibility that FBB18 and its associated DNAAFs also functions during translation of DHCα. These points are included in the discussion.

Per the suggestion, we also examined other subunits of the dyneins, which are listed in Table S1. To determine whether a subunit was likely associated with FBB18, two criteria were used: more than two peptides for each subunit were identified in the FBB18 IP; a PSM ratio for each subunit (FBB18 IP to the control) is more than two. By these criteria, we found no subunits are associated with ODA HCs. Thus, these data suggest that FBB18 and its associated DNAAFs participate in the folding of ODA HCs before a holo-complex of ODA is formed. Interestingly, we found three subunits associated with IDA, namely, DIC3/IC140, DII6/IC97 and p28. IC140 and IC97 are components of double-headed dyneins while p28 are components of dynein a, c, and d (Yamamoto et al., 2021 Cytoskeleton). Because the other components of these IDA dyneins were not enriched, we propose that FBB18 and its associated DNAAFs are involved in the folding of IDA HCs and likely these accessory components before a holo-complex of IDA dynein is formed.

These information are provided in the results and/or discussion.

6. The authors observe that loss of cfap298 leads to an increase in the IFT dynein and suggest that its folding/stability does not depend on CFAP298. The observation that no DNAAFs (including CFAP298) have been reported to adversely affect cytoplasmic or IFT dynein assembly was made previously (see ref #39) and should be acknowledged.

This ref is cited accordingly.

7. The authors state that CFAP298 participates in dynein folding in cytoplasm based on interactions with several DNAAFs and contrast this with previous proposals that it might target dyneins to cilia or stabilize them within cilia as CFAP298 was found in cilia. As the authors also demonstrate and confirm the presence of CFAP298 in isolated cilia and the cilia membrane/matrix fraction what role do they predict it plays there? Presumably not an early step in preassembly? As many DNAAFs are totally absent from cilia this is very different and although the cilia signal in Fig 3C is quite low, a very robust signal is shown in Fig 3B. Perhaps including Coomassie stained gels would allow the gel loadings to be better appreciated. I think at several places in the manuscript there is an assumption that a role in cytoplasmic folding/stability precludes involvement in other activities or locations, but without providing any data to rule them out. This can be readily dealt with by rewriting several sections to be less assertive. Considering how long it takes a ribosome to actually synthesize a dynein HC, it is also unclear how “chaperoning” per se can be readily distinguished from many other steps in the pathway to functional dyneins as they are likely cotemporaneous and may occur prior to the completion of HC synthesis.

Great points. We propose that FBB18 functions in folding/assembly of axonemal dyneins based on several evidences including reduced abundance after loss of FBB18. Because FBB18 is associated with several DNAAFs, we revised the text to say that FBB18 with its associated DNAAFs participate in the folding.

About the totally absence of DNAAFs in cilia, we are not sure how many DNAAFs are claimed that way. As far as we understand, most of them are said to be primarily or predominantly present in the cell body. In mammalian cells, most studies use immunostaining, which have low resolution for protein identification than immunoblotting. Because of the easiness of isolating cilia in Chlamy, people are able to detect proteins in cilia by immunoblotting. This detection depends on the sensitivity of the antibody, the protein amount loaded, the exposure time used and etc. For Figure 3C, if one uses short time exposure for the blot, one could not see anything in the cilia sample. For Figure 3B, that the signal is robust is basically because we wanted to see the signal and longer exposure time was used. We examined literatures to see whether any DNAAFs were examined in cilia by immunoblotting. We found that PF13, likely the first reported DNAAF, is present in cilia (Omran et al., 2008 NG). In Figure 3e of this publication (see below for the image), one can see a faint band in cilia. When the exposure time is increased, a robust signal would be expected.

We agree that folding and assembly may occur at the same time.

8. A previous study found more CFAP298 in flagella and cell bodies of several motility mutants compared to wildtype. The data provided here in Fig S2 using a different antibody show less increase and the authors say any differences are not significant (lines 200-202) but on what basis is unclear. Indeed, it does seem under some conditions that increases are actually observed. For example, in Fig S2A expt 1 there would appear to be clearly more CFAP298 in ida1 cells compared to wildtype and similarly in Fig. S2B expt 1 there is more in oda1 and oda5 cilia compared to wildtype (this may also be the case in expt 2 but the blot exposure is very dark). Also in Fig S2B, the amounts in pf20 cilia seem to be consistently much less than other strains. At the least, there does appear to be considerable variability in the signals obtained in the various mutants, which should be directly acknowledged even though the origin of the variation might be uncertain.

It was concluded from a previous publication that all motility mutants tend to accumulate FBB18. However, we could not find the loading control in that paper. For whole cell samples, though FBB18 was elevated in some mutants in one of the experiments, this, however, could not be reproduced. For the cilia samples, we agree that there are some variability. We have revised the text to reflect this.

9. Fig 3C – why is there no tubulin signal in the whole cell/cell body samples while signal is readily visible in the cell extract blots shown in Fig 1E? Again, Coomassie stained gel/blots would aid interpretation here.

We loaded equal amounts of proteins for each lane, 10 μg proteins were loaded. As microtubules are highly enriched in cilia, it is expected to observe a strong signal from cilia. When higher exposure is used, one should be able to see the tubulin signal in the cell body. This experiment was to demonstrate there is less FBB18 in cilia. If the protein is loaded less for the whole cell or cell body sample, it still supports our conclusion. Secondly, our immunostaining data also shows that FBB18 is primarily present in the cell body.

10. Also in Fig 1E, there are several non-specific bands detected by the CFAP298 antibody that are present in all cell extracts. However, in addition to CFAP298 itself, the CFAP298 antibody also detects a band at ~50 kDa in wildtype that disappears in the mutant and reappears in the rescued strain. Is this some other CFAP gene product or splice variant? Is it detected by the HA tag antibody (the WB strip shown is not large enough to tell)? Would it also be included in the IPs shown elsewhere in the paper, and how might this affect the interpretation of protein-protein interactions especially for proteins found in low abundance?

If there were splice variants, the upper blot should show this because we used FBB18 antibody for the blot. Thus, we could exclude this possibility. One of the explanations is that *fbb18-1* and wild-type cells have slight different cell morphology and physiology. *fbb18-1* forms palmelloids and short cilia, which might impact the cell physiology and thus shows a slight different protein profiles, which were picked up by the antibody that is not strictly specific for FBB18. Thus, these slight different protein bands should be unrelated to cilia. In addition, our IP is using GFP antibodies instead of FBB18 antibody. Therefore, we think this would not affect our interpretation of protein interactions.

11. Immunostaining in Fig 3D indicates a punctate pattern throughout the entire cell including where I would anticipate the chloroplast, which fills much of the cell, is located. Is this a maximal projection image or ?????, the methods are very unclear on this. Please clarify.

Regular confocal microscopy was used for the imaging. For immunostaining, membrane permeabilization agents are normally used. We have used methanol for fixing and permeabilization. During this process, the chloroplast is expected to be damaged. The cytoplasm would flow everywhere in the cell. We checked Pete Lefebvre’s work on LF1’s localization (Nguyen et al., 2005), it appears that it is also everywhere in the cell. See the image below (from Figure 6D).

12. The diagram in Fig 4A indicates that CFAP298 interacts directly with 6 different proteins. Given its rather small size, it seems unlikely this could all occur at the same time. Does binding of one or more of these components preclude binding of others? This information might significantly aid interpretation of CFAP298 function.

Excellent points, we 100% agree. This is really intriguing. Currently we do not have any satisfying explanations for this. It is likely that all these interactions are transient and a stable complex may not be formed. In the revised text, this question is raised.

Thanks again for the constructive comments and suggestions, which helps a lot to improve the manuscript. We sincerely appreciate it.

**3. Reviewer #2:** Summary. The authors use multiple complementary methods to analyze the function of CFAP298 in Chlamydomonas. The data from the insertional CFAP298-1 mutant suggests a role with the assembly of specific heavy chain outer dynein arm DHC alpha and inner dynein arm dynein b. For such function, CFAP298 interacts with many other dyneins. I have a few questions and requests for additional information that may improve the manuscript.

Thanks for the constructive comments, very appreciated. We just wanted to remind that CFAP298 is revised to FBB18 for the chlamy gene/protein in the revision per the suggestion of the Editor.

Questions

Line 135 and Figure 2C and Figure 3E. If only 23% of the transformants from the rescue had a normal phenotype, how does the level of DHC alpha and dynein b appear identical to the wild type levels?
In Chlamydomonas, a transgene along with a selection marker is transformed into the cells. Individual colonies that are grown on the selection agar plates are picked up for testing transgene expression. 23% means that 23% of colonies tested are expressing transgenes. The positive colonies or clones (or so called rescues) are used for further analysis. Thus, it is not a mixture of rescued and un-rescued cells for immunoblotting.

Line 199 and Figure 3B, S1A. Please discuss the role of CFAP298 in the flagella/cilia. Such location is not consistent with other DNAAFs. Is it possible that there is an additional transport role.
One of the advantages of Chlamydomonas is that cilia can be easily separated from the cell body, which provides us the opportunity to test the protein distribution in cilia and cell body by immunoblotting. Immunoblotting is much more sensitive than immunostaining, and allows for the detection of trace amount of proteins in cilia. In Figure 3C, when equal amounts of proteins are loaded, one can tell that FBB18 is primarily localized in the cell body. Furthermore, in Figure 3D, the immuostaining data also shows that FBB18 is primarily localized in the cell body and barely any signal is detected in cilia.

In mammalian cells, some of the DNAAFs might be claimed to be exclusively present in cilia. This is likely because immunostaining is used to detect the proteins, which has lower sensitivity. Actually as far as we understand, most DNAAFs are said to be primarily present in the cell body. We examined literature to see whether any DNAAFs were examined in cilia by immunoblotting. PF13, likely the first reported DNAAF, is deed present in cilia (Omran et al., 2008 NG). In Figure 3e of this publication (see below for the image), one can see a faint band in cilia. When the exposure time is increased, a robust signal would be expected. We analyzed the cilia samples using longer exposure for the immunoblot. It appears that cilia have decent amount of FBB18. Actually it is not when compared to the cell body form of FBB18.

The function of the trace amount of FBB18 in cilia is not known at least from the experimental data. In the previous paper (Austin-Tse et al. 2013), it was thought that FBB18 functions in cilia. This is based on the observation that the level of FBB18 in cilia of motile mutants is elevated. However, there are no protein loading control in that paper. That is why we repeated the experiments with loading controls. We could not reach the same conclusion. In addition, they did not directly compare the protein level of FBB18 in cilia versus cell body.

Thus, from our data and the above analysis, we tend to think that FBB18 does not play a major role in cilia.

Line 206 and Figure 3D. The figure 3D should be improved. The CFAP298-HA images be improved and especially brightened? It is difficult to see the puncta in green contrasted to black and observe if there is any signal in the flagella. Can the authors co-localize CFAP298 with axonemal dynein motor client cargo? Such experiments would strengthen the difference from prior published studies (ref 31) and support the IP experiments.
We bet that the images seen are from the converted files, which has bad resolution. One evidence is that the images in the supplemental files that are not converted from the editorial system are clear.

Indeed, one should not expect to see any puncta in cilia because immunoblotting (Figure 3C) shows that FBB18 is present at extremely low level in cilia compared to that in the cell body. The dynein antibodies were kindly provided to us 5 years ago by our Japanese and American colleagues. The antibodies runs low and the quality is not sufficient for immunostaining studies. It is a good point that we should keep in mind for future studies.

Lines 234-237 and Table 1. The explanation for finding more DHC alpha in the CFAP298-1 cells compared to control cells is not obvious. If CFAP298 if functioning to stabilize dynein (Line 214) would it not be likely that the target protein would be degraded? The western blot in of the mutants shows it is not detect in Fig 3E ?
To find the changes in the level of dynein in the mutant, we did a comparative proteomics with the wild-type cells. What we did is that we mixed equal amounts of proteins from mutant and the wild-type cells, one of them was labelled. Then the mixture of cells were lysed and analyzed by proteomic analysis. The dyneins from control and mutant can be distinguished by the labeling and the ratio can be calculated via the open software resource. In mammalian system, amino acids with stable isotope are usually used for labeling while here ammonium nitrate with N15 is used because algae are lower plants.

Minor points

Table 1. Please explain and add to methods how were counts normalized for each mass spec sample and how the average ratio determined.
The counts were not normalized. We used equal amounts of proteins for the mass spectrometry analysis. The raw data is analyzed by the pFind algorithm with default setting. The data from the two analyses are very similar. For example, the ratio of DHCγ in the first and second experiments are 0.99 and 1.02 respectively. The average ratio is obtained from results from the first and second experiments. To make it more clear, we have added (*fbb18*/control) underneath “Average ratio).

Line 131. Please include the site of the immunogen for the ab to CFAP298 in the main text. It is important for interpreting the data and I could not find that information in the methods or supplements.
The information for the antibody was provided in an earlier publication, which was cited in Table S1. Actually, full-length expressed protein was used as an antigen.

Fig 4A. It would be useful to have a key for solid and dashed lines. Some interactions are previously reported interactions and identified in the current manuscript, so both types of lines would be used?

The solid and dashed lines were denoted in the figure legend. Per the suggestion, both types of lines are used.

**4. Reviewer #3:** This study uses a CFAP298 null mutation in Chlamydomonas to add to biological understanding of axonemal dynein arm assembly, a complex chaperone-driven process. CFAP298 is not well understood after human disease and zebrafish studies and this report adds a useful critique of past papers and the current understanding. Defective CFAP298 is here linked to flagellar motility and length defects plus defects of both outer and inner arm dynein assembly. In constructs over-expressed on a CFAP298-null background, deletion of either the CFAP298 CC or DUF domain leads to protein instability. Over-expression of del252-277 was more useful, mimicking a reported human mutation causing PCD with dynein arm defects. This construct was less able to rescue flagella motility or ODA structures that FL. Mass spec interaction studies of CFAP298 to identify its protein network shed some fresh light on its protein partners and notably showed that CFAP298 is likely to interact with RuvBL1/2 without SPAG1 and RPAP3.
Generally speaking, this study adds information to the field, there are some additional controls that could have been done on protein experiments and some of the English grammar could benefit from review sub editing.

Thanks for the appreciation of this work and for constructive comments.

1. Have the authors looked at an alphafold model of CFAP298 and whether the model can tell something about functions of domains of the protein and mutations, in particular how it could fit into a R2TP type of complex.

Please note, in the revised version, we are using FBB18 for the Chlamy homologue of CFAP298 per the suggestion of the editor. It is an excellent suggestion. Indeed, we thought about analyzing the structure of a possible complex of FBB18-RuvBL1/2. The first thing we did was to determine whether they interact strongly. We purified the proteins and analyzed their interactions by microscale thermophoresis and found that the interaction was not that strong. In Figure 4E, only a small portion of FBB18 associates with RuvBL1/2. Thus, we think they may not form a tight and stable complex and did not perform further structural analysis.

2. Fig1B, it is unclear how exactly the gene insert affects the CFAP298 protein to create a null allele; e.g., a fusion protein? Early termination?

FBB18 antibody was made by using the whole protein as an antigen. We did not detect any possible band of FBB18 on our immunoblot of *fbb18* (Figure 1E), suggesting that the insertion may not induce early termination. As far as know, there is no studies on the mechanisms in Chamydomonas. Certainly it is an interesting issue.

3. In the case of the human mutation p.Tyr245*, nonsense mutations are generally expected to lead not to an expressed truncated protein, but to nonsense mediated decay and degraded hence no protein and in this sense the Chlamy over expressed delta252-277 may not be a good model of the mutational effect. Is there any evidence for the human mutant protein and whether it is expressed or lost from patient cells?

Excellent points! We totally agree. Based on this, we have revised the manuscript to reflect the concerns raised. As far as we know, it was not examined whether patient cells have the mutant protein or not. One thing is to notice that the patient with this mutation has less severe dynein arm defects than the other patients. Please note, we have made a mistake. p.Tyr45* described in the previous version is indeed p.Tyr264, which is revised accordingly.

4. Up to Fig3 the authors talked about delta252-277 but they don’t mention anything about this construct in Fig4. It would be interesting to know how this truncation affects protein-protein interactions shown in Fig4.

Our data show that delta252-277 appears to affect manly DHCα. As the mutation in DNAAFs often cause defects in multiple dyneins, we think this domain is unlikely involved in the interaction with other DNAAFs rather dynein heavy chains.

5. Fig 2C and 3E cilia versus cells, in the discussion what is the relevant comparison for these similar experiments. If possible, it would be useful to create a summary cartoon of the predicted effect of the loss of CFAP298 on the axonemal dyneins structures.

In the EM studies of cilia, we analyzed the loss of dynein arms in the null mutants. For the proteomic analysis, whole cell samples were used. We do not know the partition of dyneins in cilia versus cell body. It is possible that partial degradation of dynein in the cell body may not affect the dynein arms in cilia because only a portion of dyneins in the cytoplasm may be sufficient for the assembly of dynein arms in cilia.

Dynein arms have partial loss as revealed by EM and immunoblotting. We are not sure the degrees of each dynein are lost from cilia. Thus, we think it would be challenging to draw a cartoon to illustrate this.

6. Table 2 and 3 IP data – these experiments pick up a lot of interactions and some are much weaker than others without any subsequent following up of these results for most. Was an empty-YFP construct included to these experiments as a control, can this data be shown in comparison? Are synchronised cultures of Chlamydomonas being compared. The text could do more to acknowledge some of the study limitations.
Can the text clarify whether Table 2 and 3 use the same mass spec data, and how many times was this experiment done, to understand variance in the results. With no follow up coIP studies shown for most of these interactions except the PF22 and RUVBL1/2, this would strengthen the evidence.

For the IP experiments, we aim to see what DNAAFs and dyneins may associate with FBB18. The interaction of FBB18 with other DNAAFs are supported by the literatures and our new data. Most of the dyneins that are found to be associated are differentially affected by the loss of FBB18. Based on these, we did not perform a second experiment, which we thought is sufficient to illustrate the role of FBB18 in dynein preassembly. Now this information is added in the methods. We have used wild-type cells as a control. In our previous experiments using anti-YFP for IP, we never pulled down dyneins. Thus the data is believable. For our culture conditions, the cells are synchronized. The data from Table 2 and 3 are from the same experiments, which are now clearly indicated in the text.

7. Discussion – refer to the possible reasons why the difference is seen in expression levels of CFAP298 in Chlamydomonas mutants in FigS1, compared to the past report [31].

Ref 31 examined the expression of several human variants in zebrafish. In Figure S1, we expressed different truncated constructs in *fbb18* mutant of Chlamy. The expression system and the constructs used are different. The underlying mechanisms would be complex and it would be difficult to draw any conclusions beyond pure speculation.

8. Punctate staining of CFAP298 in the cytosol – have authors seen evidence that it is present in DyNAPs, if TEM was used. Ref to work of Wallingford group PMID: 33263282.

We did not examine the co-localization of FBB18 with other DNAAFs because the available dynein antibodies have low quality. We are afraid that one could not distinguish DyNAPs by TEM. Whether there are DyNAPs in Chlamy remains to be tested. In text, we have addressed this concern and the ref is cited.

9. Discussion finishes abruptly – is it suggested from this data that CFAP298 could be in a different type of R2TP like complex, or in a differently structured complex? The notion of multiple R2TO type complexes as set out in reference 16, is the author’s speculation that this is still most likely for CFAP298.

In ref #16, three R2TP-like complexes containing RuvBL1/2 are proposed: R2SP, R2T, R2SD. What we found in this work is that FBB18 could associate with RuvBL1/2 in a complex that does not have RPAP3/SPAG1 and MOT48, a PIH domain containing protein. This data suggests that R2 may function without direct association with a protein containing TPR domain or a PIH domain. In this regard, R2-FBB18 may represent a new type of R2TP-like complex.

Technical comments, typos:
- Fig1C graph, refers to cell numbers with sizes – how is this important what does it say about phenotype? This graph also needs to show the equivalent data for WT to understand what the normal spread of cell size is.

Chlamydomonas palmelloids indicate a cluster of cells encysted in a single mother cell wall as shown in Figure 1D. Cells without flagella in Chlamydomonas usually result in such phenotypes because flagella are required to secret enzymes to digest the mother cell wall after cell division. Wild-type cells which have flagella are unicellular. We described this because it was the phenotype that we observed and was another indication that the cells had flagellar defects. The data show the number of cells of different cell types instead of cell size.

- What does it mean that cells were ‘hatched from sporangia for unknown reason’ with reference to Fig1, this is not further discussed, what is the relevance?

Chlamydomonas cells divide within a mother cell wall. The mother cell wall has to be broken to release the daughter cells (hatching). Otherwise, the daughter cells will cluster to form palmelloid. When flagella of daughter cells are formed, cell wall digesting enzymes will be secreted from the flagella to digest the mother cell wall to release the daughter cells. Our data suggest that there are other mechanisms involved in hatching that is influenced by growth state. It will inform other researchers for this phenomenon in case some of them are interested in this issue for further investigation. Secondly, this phenomenon is also linked with flagellar phenotypes.

- Text on Fig1 suggests we should be seeing evidence of a 23% rescued phenotype – what data does this refer to?

In Chlamydomonas, the rescue experiments are usually performed as the following. After transformation, the cells are grown on the agar plates and each colony is picked up and further grown in large quantities in liquid medium in a flask or a microtiter plate. Each cell culture was examined for phenotype and gene expression. Not every colony that grows on the agar plate would express the transgene. The rescue percentage was obtained by the number of rescues in total number of colonies screened. This data was just to show the rescue efficiency because different genes have different rescue efficiency. It also indicate that the rescue was caused by transgene expression rather by random insertion of the transgene. Thus, this system is different from the mammalian system.

- Page 6 of text only refers to Figure 1 for motility/length evidence, this needs to refer to Fig 2 data graphs.

Revised per the suggestion.

- Fig2B why does the WT lack cross sections containing the expected 9 ODAs.

Careful observation!. In Chlamydomonas cilia, the number #1 MT doublet does not have ODA, which appears to be a unique feature of Chlamydomonas. The underlying mechanism is still unknown.

- Fig2D, these graphs really should show the equivalent cfap298-1 mutant data as well as the rescue data, for cilia length and beat frequency. Technical details seem incomplete – how many times was the experiment done, number of cells tested?

In Figure 1C, we showed the ciliary length data of the mutant. For the motility, because the majority of the mutant cells do not have cilia or very short cilia, we thought that it would not produce useful information for the motility assay. All the data were reproduced. The beating frequency was from measuring 39 cells. The ciliary length was from measuring 50 cilia. In the revised text, the information is added in the method section.

- The Fig2D data could benefit from showing movies of the rescued or non-rescued beating and length.

The length is shown in Figure 2D. Previously we thought that addition of the movie was unnecessary. Per the suggestion, we have provided the movies as Movie S1-S3.

- Page 6 first referring to CFAP298-HA, this needs to explain this is a FL HA tagged version.

We have thought that it would indicate a full-length construct. Per the suggestion, the text is revised to the following: Expression of a full-length *FBB18* tagged with HA (FBB18-HA).

- Fig3A the use of a nonspecific band as a control is clearly unusual and seems unacceptable if that protein is unidentified. What is pdf, a control? Where can we see the wildtype levels of protein, to judge the level of successful rescue?

In our opinion, using a non-specific band as a loading control would be much more accurate because all the signals come from the same loading lanes and on the same blots. In addition, studies using coomassie blue stained blot as loading control can be found elsewhere. We thought this was acceptable.

This experiment was performed using wild-type cells. Cells were deciliated and samples were taken before deciliation and at different times after deciliation. pdf is an abbreviation for pre-deflagellation. Per the suggestion of the editor, in the revised ms, it is revised as pdc, pre-deciliation, which is indicated in the legend.

In Figure 1E, we compared the level of the protein in wild-type cells and the rescue, the protein level is very similar.

- Fig3E would benefit from a normalised graph of protein levels compared to CBB.

We have done the whole cell proteomics and analyzed all the dyneins. The data are listed in Table 1, which would give an overview of all the dyneins. The immunoblotting was only using a few antibodies. Per the suggestion, we have normalized the dynein levels and provided the ratio in the figure legends. Please note, the ratios presented are slightly different from the proteomics data, which may be caused by the limitation of quantification by using immunoblotting. However, the overall trend is similar.

- Page 10 starts ‘After ascertaining that CFAP298 is a DNAAF etc’, but this is already established with human gene nomenclature DNAAF16 well established and so on. Need to indicate this is a confirmation therefore, or is it the first finding of such.

Though it was assigned as a DNAAF, the available evidences do not fully support it. For example, we have not seen evidence that FBB18 mediates the stability/folding of axonemal dyneins in the cytoplasm. Per the suggestion, we have revised the text using the word confirmation.

- Fig4B the criticised Y2H data missing key CFAP298 interactions, this seems better to be moved to supplementary data then.

The dataset does not take too much space and it would allow the readers to see all the data once and save the readers’ time.

- Table 1 data could benefit from a graph to show clearly the changed protein abundances.

Thanks for the suggestion. We figured that addition of one graph would be repetitive given the data is already shown in the table.

- Table 3 what is the relevance in R2TP complexes of the two HSP90s A, C, and the HSP70E, which have been associated to DNAAFs before.

We searched our dataset for chaperones and known DNAAFs and all of them are listed. These data would inform the readers what chaperones are identified in this study..

- In text and figures ensure DYX1C1 has the final 1 on the end, this is missing a couple of times.

Thanks, changes are made in text and figures.

- Bottom of page 12 add reference to Figure 4E here in the text.

Revised.

- Page 14 typo DNNAFs, check for a few more minor typos. Such as line 351 page 14 attractive, line 384 page 15 axonemal, line 385 loose network.

These are changed accordingly. Thanks again for all the comments and suggestions.
