# Supplementary material for: FBB18 participates in preassembly of almost all axonemal dyneins independent of R2TP complex
Source: PLoS Genet. 2022 Aug 26;18(8):e1010374. doi: 10.1371/journal.pgen.1010374 (PMC9455862; doi:10.1371/journal.pgen.1010374)
Supplement: Attachment — Submitted filename: Response to reviewers.docx [file pgen.1010374.s011.docx]

We are grateful to the Editor and reviewers for the constructive comments.

1. Response to the general comments

Please put the TG in the name of the gene on lines 257 and 779.

TG was added following the suggestions: *FBB18-YFP* is changed to *FBB18:YFP-TG* on line 257; *FBB18-HA* is changed to *FBB18:HA-TG* on line 778 (previous line 779). In addition, we have added *FBB18:HA-TG* on line 134.

Figure 3 does not have a control for the level of contamination for the cytoplasm in the cila prep.  Is the FBB18 signal just cytoplasmic contramination;? Please use the NAB antibody or PFOR antibody to ask about cytoplasmic contamination.  This is key since ciliary localization in not observed in C. elegans or human cells.

Ciliary localization of FBB18 in *Chlamydomonas* has been shown in a previous publication (see Figure 4 in Austin-Tse et al., 2013). Our data is consistent with that finding. However, per the suggestion, we have performed new experiments with anti-NAB antibody as another control. Our data showed there is no visible contamination from the cell body. The new data has replaced the previous data (Figure 3C) and the figure legend is accordingly revised.

2. Response to the reviewers’ comments

Reviewer #1: I think the authors have addressed most of my comments. However, concerning the variable amounts of FBB18 in cilia samples from various mutants, although they state they now acknowledge there is variability, the legend to the supplemental figure S2 still contains the statement “no significant changes in the abundance of FBB18………”. If one looks for example at the pf20 cilia, there is very obviously much less FBB18 than in say oda5 cilia; a similar statement can be made about wildtype vs. oda5. The lower cilia blot is very over exposed but even there the differences in samples are clear. Surely, this is significant and it is also consistent. If not, what difference would the authors consider significant? So I still think the authors are seeing consistent changed levels of FBB18 between at least some of the strains they examined.

We are sorry that the information in the legend of Figure S2 was not revised in the previous version. The abundance of FBB18 was increased in the cilia of some mutants while it was deceased or barely changed in some other mutants. Based on the comments, we have rephrased the sentences in Figure S2 legend as the following: Please note, the ciliary abundance of FBB18 varies with the mutants. We failed to observe a correlation between the ciliary abundance of FBB18 with the phenotype of impaired cilia motility.

I am also not sure I agree with the author’s point concerning the immunostaining which shows a punctate pattern throughout the cell even where the chloroplast should be. The argument is that on fixation the cytoplasm can flow into the damaged chloroplast and thus the antigen appears to be everywhere. If correct, doesn’t this negate the entire point of the experiment, which is to localize FBB18? Shouldn’t every other cytoplasmic antigen show the same pattern in this case? Perhaps a different fixation scheme using an aldehyde rather than methanol should be used to avoid this “flow”.

The experiments aim to show the presence of FBB18 in the cell body by using immunostaining. In our opinion, it still supports our conclusion even if FBB18 “flows” due to the technique used in the immunostaining. We admit that the experiment was not perfect. Unlike in mammalian cells, the autofluorescence from the chlorophylls interferes with the immunostaining signal if the chlorophylls were not extracted. Definitely, the immunostaining protocol should be improved.

Reviewer #2: Summary. The authors have submitted a revised text with additional explanations of their data. I have a few suggestions to improve comprehension of the data.

1. The writing and grammar should be improved, particularly in the new (revised) text. The abstract should especially be edited for clarity. For example, Line 22: The sentence confuses the wild type and mutant alleles. I did not see evidence that the mutation of FBB18 localizes to the cytoplasm. I suggest moving cytoplasmic localization to line 20: Here, we show that FBB18, a Chlamydomonas homologue of CFAP298, localizes to the cytoplasm and functions in the folding/stabilization…
we have re-examined the revised text and polished the writing. Indeed, we found a few mistakes. Thanks for the editing, which is very helpful. Changes have been made as suggested.

2. Results. Lines 240-252 are difficult to follow in the revised form since the reader has to move between the immunoblots in Figure 3 and Table 1 mass spec data. I suggest inserting another reference to (Table 1) at the end of the sentence referring to IFT dynein on line 249.
Sorry for this confusion. “Table 1” is inserted at the end of the sentence (line 249).

3. Line 131. Please include the immunogen used to generate the ab to FBB18 in the main text. It is important for interpreting the data and I could not find that information in the methods, figure legend or supplement. Another reviewer asked for that same information. It is not useful to only reference that information.

Sorry for our misunderstanding. The information for the antibody is now included in the method in the section of Primary antibody. The added information is as the following: Please note, a rabbit polyclonal antibody of FBB18 was raised against a full-length polypeptide and affinity purified.
